# Translating microarray data for diagnostic testing in childhood leukaemia

**DOI:** 10.1186/1471-2407-6-229

**Published:** 2006-09-26

**Authors:** Katrin Hoffmann, Martin J Firth, Alex H Beesley, Nicholas H de Klerk, Ursula R Kees

**Affiliations:** 1 Division of Children's Leukaemia and Cancer Research, Telethon Institute for Child Health Research and Centre for Child Health Research, The University of Western Australia, Perth, Australia; 2 Division of Biostatistics and Genetic Epidemiology, Telethon Institute for Child Health Research and Centre for Child Health Research, The University of Western Australia, Perth, Australia

## Abstract

**Background:**

Recent findings from microarray studies have raised the prospect of a standardized diagnostic gene expression platform to enhance accurate diagnosis and risk stratification in paediatric acute lymphoblastic leukaemia (ALL). However, the robustness as well as the format for such a diagnostic test remains to be determined. As a step towards clinical application of these findings, we have systematically analyzed a published ALL microarray data set using Robust Multi-array Analysis (RMA) and Random Forest (RF).

**Methods:**

We examined published microarray data from 104 ALL patients specimens, that represent six different subgroups defined by cytogenetic features and immunophenotypes. Using the decision-tree based supervised learning algorithm Random Forest (RF), we determined a small set of genes for optimal subgroup distinction and subsequently validated their predictive power in an independent patient cohort.

**Results:**

We achieved very high overall ALL subgroup prediction accuracies of about 98%, and were able to verify the robustness of these genes in an independent panel of 68 specimens obtained from a different institution and processed in a different laboratory. Our study established that the selection of discriminating genes is strongly dependent on the analysis method. This may have profound implications for clinical use, particularly when the classifier is reduced to a small set of genes. We have demonstrated that as few as 26 genes yield accurate class prediction and importantly, almost 70% of these genes have not been previously identified as essential for class distinction of the six ALL subgroups.

**Conclusion:**

Our finding supports the feasibility of qRT-PCR technology for standardized diagnostic testing in paediatric ALL and should, in conjunction with conventional cytogenetics lead to a more accurate classification of the disease. In addition, we have demonstrated that microarray findings from one study can be confirmed in an independent study, using an entirely independent patient cohort and with microarray experiments being performed by a different research team.

## Background

Acute lymphoblastic leukaemia (ALL) is a heterogeneous disease characterized by the presence of several subtypes that are of prognostic relevance. These subtypes can be distinguished based on immunophenotype, differentiation status, as well as chromosomal and molecular abnormalities. The identification of different ALL subtypes, the characterization of prognostic features, and the finding that ALL subtypes differ in their response to therapy has greatly facilitated the development of treatments tailored to specific subgroups [1-3]. Current National Cancer Institute (NCI) criteria for risk assignment utilise age and white blood cell counts (WBC) at diagnosis to stratify patients into standard risk (SR; 1-9.99 years of age and WBC<50,000/μl) and high risk (HR; ≥ 10 years of age or WBC ≥ 50,000/μl) [4]. In addition, several structural and numerical chromosomal abnormalities are known as independent prognostic factors. For example, the t(9;22) translocation is strongly associated with poor prognosis, whilst both t(12;21) translocations and high hyperdiploid karyotypes (>50 chromosomes) confer a favourable prognosis [5-7]. Although detection accuracies for chromosomal abnormalities can be as high as 90%, the success rate varies greatly and cytogenetic analysis remains a challenge due to the low mitotic index and poor quality of the metaphases associated with ALL [7,8]. Cytogenetic interpretation can be particularly difficult for complex karyotypes, cryptic translocations such as the *TEL-AML1 *translocation, and multiple chromosomal rearrangements that have been identified for the same locus, as is the case for chromosomal abnormalities involving the *MLL *gene. Thus, multiple complementary technologies, such as fluorescence in situ hybridization (FISH), spectral karyotyping (SKY), Southern blot analysis and RT-PCR, are often required for the accurate identification of chromosomal abnormalities and hence add to the extremely time-consuming and expensive process of cytogenetic analysis [5-7,9].

Recent advances in microarray technology have shown that subgroups of ALL as well as acute myeloid leukaemia (AML) can be accurately distinguished based on their gene expression profiles [10-16]. Two of the largest childhood ALL microarray studies published so far demonstrated the presence of distinct gene expression patterns in six known prognostic subgroups [13,14]. Using supervised learning algorithms to assign ALL samples into their respective subgroups, the study conducted at the St. Jude Children's Research Hospital achieved an overall prediction accuracy of about 96% [14]. The findings from this and other studies raised the prospect of developing a standardized diagnostic gene expression platform to enhance accurate diagnosis and risk stratification. One of the major challenges that lies ahead is how the information gained through microarray experiments can be applied to clinical diagnostics, including the issue of whether to employ microarrays themselves as a platform for testing. Here, we explored the possibility of using a small number of genes in such a test, which would allow the exploitation of quantitative reverse transcriptase polymerase chain reaction (qRT-PCR) as an alternative method for diagnostic screening. Compared to microarray technology, qRT-PCR has the advantage of being less expensive, rapid, already established in many laboratories and independent of extensive computational analysis. We examined the ALL microarray data set published by Ross *et al *[14], focusing on 104 specimens from ALL patients that represent six different subgroups defined by cytogenetic features and immunophenotypes. Using the decision-tree based supervised learning algorithm Random Forest (RF), we determined a small set of genes for optimal subgroup distinction and subsequently validated their predictive power in an independent patient cohort. We showed that only 26 genes are required to accurately discriminate the six known prognostic subgroups of paediatric ALL, a number small enough to allow their expression levels to be measured by modern qRT-PCR technology in a clinical setting.

## Methods

### Patient specimens and gene expression profiling

The study material included 47 Ficoll-Hypaque purified and cryopreserved bone marrow (BM) or peripheral blood (PB) specimens from children diagnosed with ALL at Princess Margaret Hospital for Children, Perth, Western Australia and 21 ALL cell lines [17]. A few cell lines included in the study are available from tissue banks, however the majority were derived from paediatric ALL patients treated at the same hospital. The study was approved by the Institutional Review Board and informed consent for the use of tissues for research purposes was obtained for all patients involved in this study. Standard cytogenetic and immunofluorescence analysis was performed on pre-treatment bone marrow, peripheral blood specimens and established cell lines. The average blast percentage for the specimens was 89.2% ± 11.0. Total RNA was extracted as described previously [18]. Biotinylated cRNA was prepared from 2 μg of total RNA, hybridized to Affymetrix HG-U133A oligonucleotide microarrays (Affymetrix, Santa Clara, CA) and arrays were subsequently washed, stained and scanned using a GeneArray Scanner (Agilent Technologies, Palo Alto, CA) according to Affymetrix protocols.

### Data extraction and normalization

Array images were reduced to intensity values for each probe (*cel *files) using Affymetrix MAS 5.0 software. These *cel *files were analyzed using the statistical software R 1.7.1 [19, 46]. The software, Methods for Affymetrix Oligonucleotide Arrays [20], a suite of functions for R, is available from the Bioconductor website [47]. Expression measures were extracted using robust multi-array analysis (RMA) [21] as previously described [22]. HG-U133A and HG-U133B raw data (*cel *files) from the ALL data set published by Ross and colleagues [14, 48] were processed as described above.

### Statistical analysis

Prior to analysis of the data set obtained by Ross *et al *[14], samples not belonging to either of the six ALL subgroups (Others n = 28) were removed. The remaining samples (n = 104) were used to identify subgroup-discriminating probe sets according to the parallel approach described by Ross *et al *[14], defined as the comparison of cases in one subgroup versus all other cases. First, we applied a variance filter to eliminate non-informative probe sets. We excluded all probe sets from the analysis with a fold-change <1.15 between patient subgroups and a p-value associated with this fold-change of >0.1, calculated using a permutation test (999 permutations).

Supervised analysis was then performed separately for HG-U133A and HG-U133B data and each of the subgroups with the remaining, informative probe sets using the decision-tree based algorithm Random Forest (RF, randomForest 3.4 standard settings) [23]. In brief, each RF analysis consisted of 100,000 trees and for each tree, the intrinsic RF reiterative process randomly chooses a subset of samples and probe sets for initial analysis and subsequently uses the remaining samples for testing back. Finally, all probe sets used for RF analysis are ranked according to their ability to discriminate between the groups of interest and for each sample a classification accuracy is obtained, along with a measure of confidence [24].

For the six subgroups (T-ALL, hyperdiploid >50, *E2A*-*PBX1*, *MLL*, *BCR*-*ABL*, *TEL*-*AML1*) 5707, 4284, 4490, 3815, 2385, 3660 HG-U133A probe sets and 1320, 3035, 1379, 1212, 976, 1347 HG-U133B probe sets passed the variance filter. The 1000 top-ranked probe sets for each subgroup from this initial separate analysis of HG-U133A and HG-U133B were combined (a total of 2000 discriminating probe sets per subgroup) and subjected to a second RF analysis. Subsequently, the 20 highest-ranked subgroup-discriminating probe sets were combined and assessed for their predictive performance using RF. The entire analysis was performed again, using MAS 5.0-calculated expression values as published by Ross *et al *[14] instead of data generated by RMA.

For cross validation, the samples were randomly divided into a training set (n = 79) and a test set (total n = 25, *BCR-ABL *n = 4, *E2A-PBX1 *n = 5, Hyperdiploid>50 n = 4, *MLL *n = 5, T-ALL n = 2, *TEL-AML1 *n = 5) and the analysis procedure described above, including the application of a variance filter, was performed 100 times [25,26]. For each analysis a new training and test set was chosen and discriminating probe sets were selected using the new training set.

PCA was used to visualize different discriminant analyses. In order to compare PCA scatter plots we determined a measure for the spatial separation of clusters, which describes tightness of clustering within each subgroup, as well as between subgroups. For this measure we first calculated the sum of the squared distances from each data point to the overall centre of the data (Sum Squared Total) and then the sum of the squared distances from each data point within one subgroup to the centre of its appropriate cluster (Sum Squared Within cluster). The spatial cluster separation was expressed as 1 – SSW/SST.

### Additional files

Supplementary information on the specimens and results from the statistical analyses are available as additional files. The primary data are available from ArrayExpress under the accession number E-TABM-125 according to MIAME guidelines [49].

## Results

### Confirmation of discrimination between prognostic ALL subtypes

**Figure 1:**
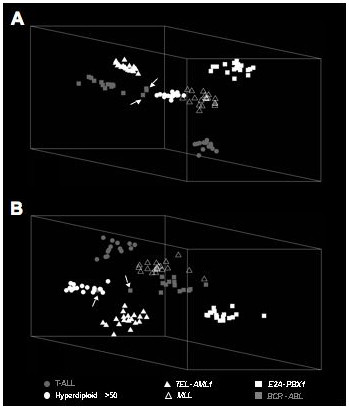
**ALL subtype distinction based on discriminating genes identified by RMA/RF**. Gene expression profiles from 104 paediatric ALL specimens were analyzed using unsupervised Principle Component Analysis (PCA). Shown are three-dimensional scatter plots of all cases using PCA with the top discriminating probe sets identified by RMA/RF. (A) Three-dimensional scatter plot of a PCA using the top 20 subgroup-discriminating probe sets (120 probe sets). (B) Three-dimensional scatter plot of a PCA using the top 5 subgroup-discriminating probe sets (30 probe sets). Arrows mark two *BCR*-*ABL*-expressing samples known to contain a *BCR*-*ABL *translocation and a hyperdiploid (>50 chromosomes) karyotype.

A study published by Ross and colleagues [14] reported the discrimination of six prognostic ALL subgroups based on 120 probe sets, using artificial neural network (ANN) as supervised learning algorithm. Comparable results were also reported when the authors used other supervised learning algorithms for classification, such as support vector machine (SVM) and *k*-nearest neighbours (*k*-NN). We opted to use a different method for analysis, comprising of RMA as data extraction method and the supervised learning algorithm RF to identify subgroup-discriminating probe sets (RMA/RF). Mirroring the analysis strategy applied by Ross *et al *[14], we compared all samples within one subgroup against all other samples (termed "parallel approach" by the authors), and identified the top 20 discriminating probe sets for each of the six subgroups (see Materials and Methods for a more detailed description of the analysis). The number of samples representing each of the six subgroups ranged from 14–20 (Table 1). RF classification with these top-ranked 120 discriminating probe sets (20 probe sets for each of the subgroups) achieved accurate discrimination of all subgroups, with the exception of two apparent misclassifications in the *BCR-ABL *subgroup (Table 1). However, these two cases are known to exhibit a *BCR-ABL *translocation as well as a hyperdiploid karyotype [14]. Overall, RF analysis achieved a slightly higher prediction accuracy of 98.1% compared to 96.4% obtained using ANN [14]. The discrimination of the six ALL subtypes based on 120 probe sets is further illustrated in Figure 1A using PCA.

### Comparison of prediction accuracies

To be able to directly compare the prediction accuracies accomplished using RF versus ANN, we implemented the cross validation procedure described by Ross *et al *[14] and divided the samples into a training set (n = 79) and a test set (n = 25, see Materials and Methods for details and Table 1). After the implementation of this cross validation, the overall prediction accuracy achieved by RF was 98.2% compared to 96.4% obtained using ANN (Table 1). As in the previous RF analysis, the classification accuracy was virtually 100% for five of the subgroups. The only apparent misclassifications occurred in the *BCR-ABL *subgroup due to the same two samples showing the *BCR-ABL *translocation and a hyperdiploid karyotype. In contrast, the cross validation performed by Ross *et al *[14] using ANN resulted in additional misclassifications of samples from the hyperdiploid and the *TEL-AML1 *subgroup, with prediction accuracies of 95% and 96%, respectively. Importantly, comparable prediction accuracies of 98.1% and 98.2% were obtained with the initial RF analysis and the cross validation RF analysis, indicating that RF is less prone to over fitting the data, which is a common problem associated with most other supervised learning algorithms. These results therefore verified our analysis approach and validated RF as a suitable alternative supervised learning algorithm for the analysis of oligonucleotide array data.

### RMA/RF analysis identifies novel discriminator genes for ALL subgroups

An important step in the analysis of microarray data is the selection of a set of discriminators that achieve optimal classification. To assess whether different analysis approaches would generate different lists of discriminating genes, we compared the top 120 discriminators for the six ALL subgroups identified by RF in the present study to those identified by Ross *et al *[14] who used a chi-square metric. Surprisingly, only 35–65% of probe sets were commonly identified in the two analyses (see Additional files 1, 2, 5). The highest level of concurrence was observed for the T-ALL,*E2A-PBX1 *and *TEL-AML1 *subgroups, with 65% of probe sets identified by both analyses. In contrast, most discrepancies were found for the hyperdiploid subgroup, with only 35% of probe sets identified in both analyses. Since some genes are represented by multiple probe sets, we subsequently compared the number of genes that had been determined to be subgroup discriminators. As expected, similar findings were obtained; 35–71.4% of genes were commonly identified in both analyses, although a higher level of agreement was observed for some subgroups (see Additional files 1, 2, 5). Interestingly, we generally observed lower average fold-changes and expression levels for discriminating genes selected by RF, compared to the analysis performed by Ross *et al *[14] (see Additional file 1). The lowest fold-changes in expression levels were detected for genes defining the hyperdiploid subgroup, a finding that agrees with the observations made by Ross *et al *[14].

We hypothesized that the relatively low representation of common genes in both analyses might be due to either the different approaches used for data extraction (RMA versus MAS 5.0), the methods used for feature selection (RF versus chi-square), or a combination of both. To address this issue, we repeated the entire analysis with the expression values generated by MAS 5.0 as published by Ross *et al *[14]. This analysis identified a third set of discriminators which captured around 65% of the probe sets identified by either RMA/RF analysis or the analysis performed by Ross *et al *[14] (data not shown). These results clearly demonstrate that different approaches used for data extraction and selection of discriminating genes can lead to the identification of different sets of class discriminating genes.

### Accurate classification of ALL subtypes using 26 genes

**Figure 2:**
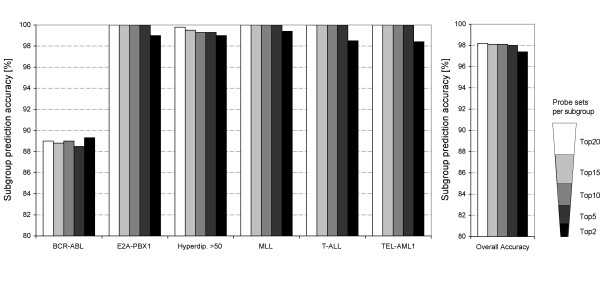
**A small set of genes accurately predicts six ALL subgroups**. Comparison of average prediction accuracies obtained with the top 20, top 15, top 10, top 5 and top 2 subgroup-discriminating probe sets identified by RMA/RF after cross validation (see Supplemental Document, Table S3). For each cross validation analysis (n = 100) a new training (n = 79) and test set (n = 25) was chosen and discriminating probes sets were selected using the new training set. Shown are prediction accuracies for individual ALL subgroups (main panel) and the overall prediction accuracies (panel on the right).

The reduction of discriminators to a small set of genes is a prerequisite for a diagnostic test that could easily be performed in a clinical setting. We addressed this issue firstly by assessing whether the number of subgroup discriminating genes could be reduced. Secondly, we determined whether reduced numbers of discriminator genes would still yield accurate class assignment in a cross validation procedure (see Materials and Methods for details). This analysis was performed with a total of 90, 60, 30 and 12 probe sets (the top 15, top 10, top 5 and top 2 probe sets per subgroup, respectively). The results of this analysis are summarized in Figure 2 (see Additional file 3) and showed that accurate discrimination of the six ALL subgroups can indeed be achieved with a reduced number of probe sets. Comparable average prediction accuracies of 98.1%, 98.1% and 98% were obtained with 90, 60 and 30 probe sets, while the reduction to 12 probe sets resulted in a slightly lower average prediction accuracy of 97.4% (Figure 2). Importantly, the levels of accuracy were very similar to the prediction accuracy achieved using 120 probe sets, and included the apparent misclassification of the two samples known to exhibit a *BCR-ABL *translocation and a hyperdiploid karyotype. Using either 90, 60 or 30 probe sets, additional misclassification occurred with very low frequencies (3.2–11.5%) for two individual specimens with a hyperdiploid karyotype and one *BCR-ABL*-expressing ALL. The reduction to only 12 probe sets resulted in further misclassification of cases belonging to all subgroups, again with low frequencies of 3.4–20.8% for individual specimens (Figure 2 and see Additional file 3). Therefore, 30 probe sets representing the top 5 probe sets for each of the six subgroups and a total of 26 genes can be used for accurate discrimination of six prognostic ALL subgroups (Table 2), which is further illustrated by a PCA scatter plot shown in Figure 1B. Importantly, we observed no differences in PCAs using either 120 or 30 probe sets, since the same degree of spatial cluster separation (see Materials and methods for details) was found in both analyses.

### Discriminators identified by RMA/RF accurately predict prognostic ALL subtypes in an independent data set

A fundamental requirement for the development of diagnostic tests is that the genes identified as discriminators in a particular study can be shown to be generally applicable. Thus, we determined whether the discriminator genes identified in the present study using RMA/RF were able to distinguish between the prognostic subgroups in our own cohort of 47 ALL patient specimens and 21 cell lines, representing the six ALL subgroups (Table 3 and see Additional file 4). We assessed the gene expression profiles for this independent cohort using Affymetrix HG-U133A microarrays. For this reason the entire analysis of the data set published by Ross and colleagues [14] described above was repeated, this time however using the HG-U133A data only. The resulting 120 probe sets were then applied to a RF analysis and the RF trained on the Ross data set was used for the classification of our 68 ALL specimens. This analysis revealed that the majority of samples in our data set were correctly classified into their respective subgroups, with an overall prediction accuracy of 92.6% (Table 3). The misclassifications included 4 cases with a hyperdiploid karyotype. Based on their gene expression profiles these samples were classified as *BCR-ABL*-positive in three cases and as *MLL*-positive in one case. In addition, one T-ALL sample was wrongly classified as *MLL *leukaemia. Importantly, when we reduced the number of discriminators to the top 30 probe sets, the RF trained on the Ross data set again classified our 68 ALL specimens with high accuracy (89.7%). This time, all samples of the T-ALL, *BCR-ABL*, *E2A-PBX1 *and *TEL-AML1 *subgroups were accurately classified, while misclassifications occurred only for cases of the hyperdiploid and the *MLL *subgroup (Table 3). The combined results clearly demonstrated that the discriminator genes identified by RMA/RF are generally applicable and are able to distinguish six prognostic subtypes of paediatric ALL in an independent data set.

## Discussion

The objective of many microarray studies is the improvement of diagnosis with the aim of accurately assigning patients into specific risk categories that facilitate risk-adapted therapy. Recent studies have demonstrated the great potential of gene expression profiling for the classification of clinically relevant subtypes of paediatric leukaemia [11-16]. The results from these studies are promising and suggest that standardized gene expression-based diagnostic tests can provide at least equivalent, if not superior diagnostic accuracy compared to conventional analysis methods. To critically assess whether findings from gene expression profiling in paediatric ALL could be adapted to diagnostic tests we have asked a set of fundamental and very specific questions: 1) Can array data be successfully applied to independent patient data, i.e. are microarray findings robust and generally applicable? 2) Is the selection of discriminating genes governed by the approach chosen for the analysis of microarray data and if so, is the selection of a different set of genes critical for accurate class assignment? 3) Is it possible to drastically reduce the number of discriminating genes without compromising the predictive performance? To answer these questions we chose the leukaemia microarray data set published by Ross *et al *[14] and re-analyzed the data using RMA [20,21] for data extraction and normalization and RF [23,27] as a supervised learning algorithm for the selection of informative genes and class assignment.

To address the first question we used our RMA/RF analysis approach to identify the top 20 discriminating probe sets for each of the six ALL subgroups represented in this data set. It is important to note that the patient cohort studied by Ross and colleagues [14] was purposefully chosen to represent all six subgroups in almost equal numbers. To independently test the discriminators identified by RMA/RF in a less "idealized" cohort, we used our own microarray data set, obtained by testing 68 paediatric ALL specimens. This analysis validated the top subgroup-discriminating genes identified by RMA/RF in an independent cohort of ALL specimens achieving overall prediction accuracies of up to 92.6%. Since our specimens were assessed using HG-U133A arrays, several top-ranked discriminators represented on the HG-U133B array were not included and this may have accounted for a less precise classification of some samples. This is particularly exemplified in case of the hyperdiploid subgroup where one of the original top five discriminators is not present on the HG-U133A array. Similarly, the only misclassification of a case with *MLL *rearrangements might be due to the highest-ranked discriminator not being part of the HG-U133A array. Despite this, our findings clearly demonstrate that the genes identified by RMA/RF as discriminators of the six ALL subgroups are robust and can be applied to an independent cohort of patients. Importantly, this set of genes accurately classified samples from an independent cohort that was obtained from a different institution, in which subgroups were not artificially represented in equal numbers, and the performance was not affected by laboratory-specific differences in terms of sample handling and data generation.

Unexpectedly, using our RMA/RF analysis we found a high degree of discrepancy between genes identified as most important discriminators for the six ALL subgroups compared to those identified in the study published by Ross *et al *[14]. Only 35–65% of probe sets and 35–71.4% of genes were commonly identified in both analyses. This finding highlights the existence of a large number of genes with the potential to discriminate between subtypes of paediatric ALL. Thus, the selection of discriminators, critical for achieving the most accurate classification and the design of diagnostic tests, seems to be dependent on the chosen analysis approach. While many microarray studies report that similar classifications are obtained with different supervised learning algorithms [13,14,28,29], so far little attention has been paid to this critical aspect of selecting discriminating genes [30-32]. Not surprisingly, we found the highest degree of variability for discriminators identified for the cases with more than 50 chromosomes. Only 7 of the top 20 discriminating genes were common between our analysis and the analysis conducted by Ross *et al *[14]. These discrepancies coincide with relatively low expression levels and particularly low fold-changes observed for the genes defining this subgroup, most likely reflecting the documented heterogeneity of this subgroup. Interestingly, the top-ranked gene *PHB *has not previously been identified as an important discriminator for this subgroup. Although the precise function of *PHB *has yet to be clarified, it has been found to play a role in several cellular processes, such as proliferation and apoptosis [33]. Other prominent subgroup-discriminating genes identified by RMA/RF were *ABL1 *for the *BCR*-*ABL *subgroup, and several B cell-specific genes with very low expression levels in T-ALL samples, including the transcription factor *EBF, PAX5*, a potential downstream target of *EBF *[34], and the transcription factor *TFEB*. Furthermore *WNT16*, a downstream target of the E2A-Pbx1 fusion protein [35], was found to be the second most important discriminator for cases with *E2A*-*PBX1 *rearrangements. The results presented here highlight that the selection of genes that distinguish best between ALL subgroups is strongly influenced by the methods used to analyze gene expression profiles, and this in turn may have profound implications for clinical applications. While RMA has more recently become a popular choice as data extraction method [20,21], only few studies have reported the use of RF as a supervised learning algorithm [23,27,36]. RF is a decision tree-based algorithm and has been proposed as particularly suitable for the high dimensionality of microarray data sets. Comparisons with other commonly used supervised learning algorithms have shown that the RF algorithm constructs far more precise classification rules [23]. Besides improved prediction accuracies, a reduction in the number of genes required for classification has also been reported when using decision tree-based methods [27].

Another critical issue that remains to be addressed is the optimal platform for a diagnostic test to measure gene expression profiles, i.e. low-density custom microarrays or PCR-based assays. Many studies, including our own, have shown that expression levels determined by microarray can accurately be reproduced by qRT-PCR [13,22,37,38]. Compared to microarrays, qRT-PCR technology has the advantage of being readily available in most laboratories, being more cost-efficient and not involving extensive statistical and computational data analysis. However, a qRT-PCR-based diagnostic platform would require the drastic reduction in the number of genes measured. The comprehensive cross-validation procedures performed in this study revealed that as few as 30 probe sets are sufficient to achieve accurate class assignment. In contrast, a previous study has reported that a single gene could identify T-ALL and *E2A*-*PBX1 *cases, while 7–20 genes were needed to predict each of the other four classes [13]. The 30 probe sets determined as requirement for accurate class prediction in our study represent 26 genes, a number that could easily be analyzed in a routine qRT-PCR test. Remarkably, only eight of these 26 genes were listed among the top 5 subgroup-defining probe sets of the study published by Ross *et al *[14]. It is foreseeable that further optimization towards developing a generic and robust classifier will lead to an even further reduction in the number of discriminators required to predict some subgroups, while additional discriminators may be needed to detect distinct subtypes within the *MLL *subgroup [16,39]. Furthermore, the inclusion of additional translocation-specific assays should enhance the accurate classification of cases expressing *BCR-ABL*.

Using our RMA/RF approach, RF discriminant analysis for the six ALL subgroups using 120 probe sets achieved very high average prediction accuracies of 98.2%. These accuracies were slightly higher than the previously reported average prediction accuracy of 94.6% [14], which included misclassification of cases with more than 50 chromosomes and *TEL*-*AML1 *rearrangements. In contrast, our analysis classified these specimens with virtually 100% accuracy. *TEL*-*AML1 *rearrangements and a hyperdiploid karyotype with more than 50 chromosomes represent two of the most frequent genetic abnormalities, found in 22% and 25%, respectively, of children diagnosed with ALL [3]. Given their prognostic significance, the correct identification of these two subgroups is of great importance. The presence of either of these features in paediatric ALL is significantly associated with a favourable prognosis [9,40-43]. Importantly, because the *TEL*-*AML1 *translocation is undetectable by conventional cytogenetic analysis, more sophisticated molecular techniques, such as FISH are required to confirm the presence of this fusion gene [6,7]. Hence, our results further emphasize the advantage of using gene expression profiles to determine this prognostically important ALL subgroup.

## Conclusion

In summary, we have demonstrated that microarray findings from one study can be confirmed in an independent study, using an entirely independent patient cohort and with microarray experiments being performed by a different research team. In this study we have excluded discrepancies due to different microarray platforms and our results argue against recent criticism that gene expression profiling may not be robust enough to be useful for clinical application [44,45]. The future challenge towards better risk stratification includes the refinement of prognostic marker genes that are associated with outcome. Our finding that only 26 genes are needed for the classification of six ALL subtypes supports the feasibility of qRT-PCR technology for standardized diagnostic testing in paediatric ALL and should, in conjunction with conventional cytogenetics lead to a more accurate classification of the disease.

## Competing interests

The author(s) declare that they have no competing interests.

## Authors' contributions

KH was responsible for designing the study, analysing, collating, and interpreting the data, and preparing the manuscript. MJF carried out the statistical analysis. AHB, MJF and NHdK assisted with data analysis, experimental design, and data interpretation. URK supervised all aspects of the study and preparation of the manuscript. All authors read and approved the final manuscript.

## Pre-publication history

The pre-publication history for this paper can be accessed here:



## Supplementary Material

Additional file 1 Table S1: Fold-change and mean expression of top 20 discriminating probes per subgroup identified by RMA/RF. Click here for file

Additional file 2 Table S2: Common probes sets and genes within the top 20 discriminators per subgroup identified by RMA/RF and Ross *et al. *(parallel format). Click here for file

Additional file 3 Table S3: Average classification accuracy (100 cross validations) of test set samples (n = 25) using the top 20, 15, 10, 5 and 2 probe sets per subgroup identified by RMA/RF. For each analysis a new training and test set was chosen and discriminating probe sets were identified using the new training set. Click here for file

Additional file 4 Table S4: Independent data set of ALL specimens used for verification of subtype classification (n = 68). Click here for file

Additional file 5 Figure S1: Identification of different subgroup-discriminating genes. Probe sets and genes that were commonly identified as subgroup-discriminators using RMA/RF and as reported by Ross *et al. *(2003). The top 20 discriminators for each of the six ALL subgroup were compared. The percentage of common probe sets is represented by light grey bars, while the percentage of common genes is indicated by dark grey bars. Click here for file

## Figures and Tables

**Table 1:** Comparison of prediction accuracies achieved by RMA/RF and ANN (Ross *et al*., 2003).

	**RMA/RF**	**RMA/RF^‡^**	**Ross *et al.*^†^**
**Analysis method**	RF	RF	ANN
**Samples in test set**	104	25	25

***BCR*-*ABL *accuracy (samples)**	86.7 % (15)*	89 % (4)*	87.5 % (4)*
***E2A*-*PBX1 *accuracy (samples)**	100 % (18)	100 % (5)	100 % (5)
**Hyperdip>50 accuracy (samples)**	100 % (17)	99.8 % (4)	95 % (4)
***MLL *accuracy (samples)**	100 % (20)	100 % (5)	100 % (5)
**T-ALL accuracy (samples)**	100 % (14)	100 % (2)	100 % (2)
***TEL*-*AML1 *accuracy (samples)**	100 % (20)	100 % (5)	96 % (5)

**Overall accuracy**	98.1 %	98.2 %	96.4 %

*By cytogenetic analysis both cases that led to apparent inaccuracies are known to show the presence of *BCR*-*ABL *and >50 chromosomes.

‡ Average prediction accuracies from 100 independent analyses. For each analysis a new training and test set was chosen and discriminating probe sets were selected using the new training set.

† Average prediction accuracies from 10 independent analyses. For each analysis a new training and test set was chosen and discriminating probe sets were selected using the new training set.

**Table 2:** Top 30 probe sets identified by RMA/RF that are required for accurate classification.

**Subgroup**	**Probe ID**	**Gene**	**Rank RMA/RF**	**Rank Ross *et al*^†^**	**Expression above/below mean**	**Fold change**
*BCR*-*ABL*	201906_s_at	*CTDSPL*	1	17	Above	2.5
	209365_s_at*	*ECM1*	2	3	Above	5.0
	210830_s_at*	*PON2*	3	2	Above	5.7
	202123_s_at	*ABL1*	4	76	Above	2.0
	222154_s_at	*DNAPTP6*	5	13	Above	6.9
						
*E2A*-*PBX1*	212148_at*	*PBX1*	1	4	Above	29.6
	221113_s_at	*WNT16*	2	18	Above	6.2
	205253_at*	*PBX1*	3	3	Above	36.1
	230306_at^•^	*MGC10485*	4	11	Above	5.9
	225483_at^•^	*MGC10485*	5	8	Above	8.2
						
Hyperdipl.>50	200659_s_at	*PHB*	1	-	Above	2.8
	216071_x_at*	*TNRC11*	2	1	Above	1.7
	208598_s_at	*UREB1*	3	12	Above	1.6
	226875_at^•^	*DOCK11*	4	35	Above	2.3
	200057_s_at	*NONO*	5	-	Above	1.5
						
*MLL*	226939_at*^•^	*CPEB2*	1	1	Above	5
	219463_at*	*C20orf103*	2	2	Above	15.2
	204069_at*	*MEIS1*	3	3	Above	10
	218847_at	*IMP-2*	4	24	Above	7
	201152_s_at	*MBNL1*	5	23	Above	2.2
						
T-ALL	213539_at	*CD3D*	1	17	Above	28.3
	227646_at^•^	*EBF*	2	23	Below	24.6
	217147_s_at	*TRIM*	3	20	Above	10.1
	229487_at^•^	*EBF*	4	24	Below	20.4
	50221_at	*TFEB*	5	-	Below	2.2
						
*TEL*-*AML1*	206231_at	*KCNN1*	1	15	Above	3.2
	241505_at*^•^	EST	2	4	Above	5.8
	206033_s_at	*DSC3*	3	12	Above	8.8
	214110_s_at	EST	4	-	Above	2.2
	206032_at	*DSC3*	5	11	Above	4.8

† The ranking refers to the top 100 subgroup-discriminating probe sets identified by Ross *et al*. using the parallel analysis format.

* Probe sets/genes identified as the top 5 subgroup-discriminators in both analyses, RMA/RF and Ross et al. (parallel format).

• Probe sets that are represented on the HG-U133B array.

**Table 3:** Analysis of independent ALL patient cohort (n = 68). Confirmation of discriminators identified by RMA/RF using HG-U133A array data.

Subgroup*	**Number of specimens**	**Correct classification**		**Classification accuracy**	
		**Top 120 probe sets**	**Top 30 probe sets**	**Top 120 probe sets**	**Top 30 probe sets**

***BCR-ABL***	3	3	3	100%	100%
***E2A*-*PBX1***	3	3	3	100%	100%
**Hyperdip>50**	17	13	11	76.5%	64.7%
***MLL***	7	7	6	100%	85.7%
**T-ALL**	37	36	37	97.3%	100%
***TEL*-*AML1***	1	1	1	100%	100%

**Total**	68	63	61	92.6%	89.7%

* Established by conventional cytogenetic and immunophenotype analysis.
