# Supplementary material for: Translating microarray data for diagnostic testing in childhood leukaemia
Source: BMC Cancer. 2006 Sep 26;6:229. doi: 10.1186/1471-2407-6-229 (PMC1609180; doi:10.1186/1471-2407-6-229)
Supplement: Additional file 1 — Table S1: Fold-change and mean expression of top 20 discriminating probes per subgroup identified by RMA/RF. [file 1471-2407-6-229-S1.doc]

**Additional file 1**

**Table S1:** Fold-change and mean expression of top 20 discriminating probes per subgroup identified by RMA/RF.

| **Probe Set ID** | **RMA/RF rank** | **Gene Symbol** | **Mean expression subgroup** | **Mean expression all other** | **Fold change** | **Rank Ross *et al*.^†^** |
| --- | --- | --- | --- | --- | --- | --- |
| **BCR-ABL** |  |  |  |  |  |  |
| 201906_s_at | 1 | CTDSPL | 43.4 | 17.5 | 2.5 | 17 |
| 209365_s_at | 2 | ECM1 | 493.8 | 98.7 | 5.0 | 3 |
| 210830_s_at | 3 | PON2 | 44.0 | 7.7 | 5.7 | 2, 1* |
| 222154_s_at | 5 | DNAPTP6 | 244.9 | 35.4 | 6.9 | 13, 16*, 54* |
| 201876_at | 6 | PON2 | 38.2 | 7.8 | 4.9 | 1, 2* |
| 204430_s_at | 8 | SLC2A5 | 157.3 | 38.9 | 4.0 | 11, 14* |
| 200953_s_at | 10 | CCND2 | 476.3 | 132.2 | 3.6 | 12, 10* |
| 201310_s_at | 12 | C5orf13 | 162.6 | 380.4 | 2.3 | 8 |
| 212242_at | 13 | TUBA1 | 116.2 | 44.4 | 2.6 | 5 |
| **202123_s_at** | **4** | **ABL1** | **116.5** | **57.4** | **2.0** | **76** |
| **219218_at** | **7** | **FLJ23058** | **46.2** | **142.0** | **3.1** | **-** |
| **223741_s_at** | **9** | **TTYH2** | **24.9** | **12.8** | **1.9** | **-** |
| **218013_x_at** | **11** | **DCTN4** | **92.3** | **26.7** | **3.5** | **55, 6*** |
| **212298_at** | **14** | **NRP1** | **38.8** | **7.0** | **5.6** | **22, 53*** |
| **227182_at** | **15** | **MGC26847** | **105.8** | **30.1** | **3.5** | **32** |
| **210510_s_at** | **16** | **NRP1** | **20.3** | **11.9** | **1.7** | **22*, 53*** |
| **201309_x_at** | **17** | **C5orf13** | **67.6** | **135.6** | **2.0** | **8*** |
| **201904_s_at** | **18** | **CTDSPL** | **27.3** | **15.5** | **1.8** | **17*** |
| **213075_at** | **19** | **LOC169611** | **46.0** | **17** | **2.7** | **43** |
| **201438_at** | **20** | **COL6A3** | **76.8** | **13.6** | **5.6** | **-** |
| **E2A-PBX1** |  |  |  |  |  |  |
| 212148_at | 1 | PBX1 | 349.6 | 11.8 | 29.6 | 4, 3*, 5* |
| 221113_s_at | 2 | WNT16 | 78.7 | 12.7 | 6.2 | 18, 20* |
| 205253_at | 3 | PBX1 | 258.1 | 7.2 | 36.1 | 3, 4*, 5* |
| 230306_at | 4 | MGC10485 | 131.7 | 22.1 | 5.9 | 11, 8* |
| 225483_at | 5 | MGC10485 | 557.3 | 68.3 | 8.2 | 8, 11* |
| 212151_at | 6 | PBX1 | 556.7 | 24.0 | 23.2 | 5, 3*, 4* |
| 35974_at | 7 | LRMP | 745.5 | 127.1 | 5.9 | 13, 2*, 100* |
| 38340_at | 8 | HIP1R | 199.1 | 76.9 | 2.6 | 14, 54* |
| 201695_s_at | 10 | NP | 367.1 | 92.7 | 4.0 | 1 |
| 212371_at | 12 | PNAS-4 | 195.2 | 75.2 | 2.6 | 6 |
| 208644_at | 16 | ADPRT | 663.2 | 260.1 | 2.5 | 17 |
| 204674_at | 17 | LRMP | 1279.6 | 254.6 | 5.0 | 2, 13*, 100* |
| 206028_s_at | 20 | MERTK | 53.6 | 8.4 | 6.4 | 15 |
| **205769_at** | **9** | **SLC27A2** | **21.3** | **6.9** | **3.1** | **45** |
| **211373_s_at** | **11** | **PSEN2** | **21.3** | **8.6** | **2.5** | **-** |
| **206304_at** | **13** | **MYBPH** | **39.4** | **19.4** | **2.0** | **-** |
| **46665_at** | **14** | **SEMA4C** | **38.9** | **20.1** | **1.9** | **-** |
| **202796_at** | **15** | **SYNPO** | **63.2** | **20.5** | **3.1** | **35** |
| **218087_s_at** | **18** | **SORBS1** | **23.9** | **5.6** | **4.2** | **65** |
| **209558_s_at** | **19** | **HIP1R** | **204.6** | **65.4** | **3.1** | **54, 14*** |
| Hyperdiploid >50 |  |  |  |  |  |  |
| 216071_x_at | 2 | TNRC11 | 140.7 | 83.8 | 1.7 | 1 |
| 208598_s_at | 3 | ---- | 299.0 | 187.2 | 1.6 | 12 |
| 205324_s_at | 7 | FTSJ1 | 109.8 | 55.2 | 2.0 | 6 |
| 201899_s_at | 9 | UBE2A | 150.9 | 82.1 | 1.8 | 7, 15* |
| 218757_s_at | 15 | UPF3B | 120.0 | 52.8 | 2.3 | 2 |
| 219485_s_at | 18 | PSMD10 | 159.8 | 76.1 | 2.1 | 17 |
| 202371_at | 19 | FLJ21174 | 69.2 | 21.8 | 3.2 | 5 |
| **200659_s_at** | **1** | **PHB** | **188.5** | **68.1** | **2.8** | **90*** |
| **226875_at** | **4** | **DOCK11** | **202.2** | **86.1** | **2.3** | **35** |
| **200057_s_at** | **5** | **NONO** | **1046.8** | **710.4** | **1.5** | **-** |
| **200738_s_at** | **6** | **PGK1** | **602.6** | **336.3** | **1.8** | **33, 72*** |
| **242794_at** | **8** | **MAML3** | **75.7** | **24.3** | **3.1** | **22** |
| **200642_at** | **10** | **SOD1** | **645.3** | **286.5** | **2.3** | **32** |
| **211342_x_at** | **11** | **---** | **155.9** | **89.3** | **1.7** | **23** |
| **208117_s_at** | **12** | **FLJ12525** | **128.7** | **85.8** | **1.5** | **63** |
| **202214_s_at** | **13** | **CUL4B** | **148.9** | **88.9** | **1.7** | **21** |
| **200600_at** | **14** | **MSN** | **606.1** | **335.8** | **1.8** | **70** |
| **212419_at** | **16** | **FLJ90798** | **26.8** | **14.0** | **1.9** | **24** |
| **203746_s_at** | **17** | **HCCS** | **77.6** | **52.1** | **1.5** | **27*** |
| **204045_at** | **20** | **TCEAL1** | **73.8** | **43.2** | **1.7** | **62** |

**Table S1:** continued.

| Probe Set ID | **RMA/RF rank** | **Gene Symbol** | **Mean expression subgroup** | **Mean expression all other** | **Fold change** | **Rank Ross *et al*. ^†^** |
| --- | --- | --- | --- | --- | --- | --- |
| **MLL** |  |  |  |  |  |  |
| 226939_at | 1 | CPEB2 | 66.2 | 12.0 | 5.5 | 1, 31* |
| 219463_at | 2 | C20orf103 | 848.2 | 55.9 | 15.2 | 2 |
| 204069_at | 3 | MEIS1 | 100.1 | 10.0 | 10.0 | 3 |
| 205821_at | 6 | KLRK1 | 336.1 | 70.5 | 4.8 | 15 |
| 231899_at | 7 | KIAA1726 | 25.5 | 4.8 | 5.3 | 9 |
| 203837_at | 8 | MAP3K5 | 20.6 | 8.2 | 2.5 | 4, 29* |
| 205899_at | 11 | CCNA1 | 55.1 | 9.0 | 6.2 | 16 |
| 226415_at | 13 | KIAA1576 | 354.2 | 23.4 | 15.1 | 5 |
| 201153_s_at | 15 | MBNL1 | 386.8 | 170.0 | 2.3 | 11, 8*, 23*, 33*, 38* |
| 235879_at | 17 | MBNL1 | 113.6 | 28.7 | 4.0 | 8, 11*, 23*, 33*, 38* |
| 211066_x_at | 18 | PCDHGC3 | 252.3 | 78.4 | 3.2 | 19, 14*, 17*, 20* |
| **218847_at** | **4** | **IMP-2** | **144.5** | **20.5** | **7.0** | **24** |
| **201152_s_at** | **5** | **MBNL1** | **652.4** | **294.8** | **2.2** | **23, 8*, 11*, 33*,38*** |
| **206099_at** | **9** | **PRKCH** | **42.5** | **89.6** | **2.1** | **27*** |
| **205726_at** | **10** | **DIAPH2** | **31.2** | **15.6** | **2.0** | **-** |
| **221676_s_at** | **12** | **CORO1C** | **180.3** | **56.8** | **3.2** | **53, 6*** |
| **203836_s_at** | **14** | **MAP3K5** | **38.6** | **16.1** | **2.4** | **29, 4*** |
| **202934_at** | **16** | **HK2** | **74.7** | **34.3** | **2.2** | **-** |
| **212135_s_at** | **19** | **ATP2B4** | **84.9** | **184.6** | **2.2** | **21, 51*** |
| **221286_s_at** | **20** | **PACAP** | **118.1** | **270.8** | **2.3** | **44** |
| T-ALL |  |  |  |  |  |  |
| 213539_at | 1 | CD3D | 1542.7 | 54.4 | 28.3 | 17 |
| 217147_s_at | 3 | TRIM | 71.4 | 7.0 | 10.1 | 20 |
| 204670_x_at | 6 | HLA-DRB3 | 84.5 | 796.1 | 9.4 | 5, 9*, 11*, 36* |
| 210982_s_at | 7 | HLA-DRA | 82.5 | 1755.7 | 21.3 | 14, 10* |
| 208894_at | 8 | HLA-DRA | 82.5 | 1850.4 | 22.4 | 10, 14* |
| 209619_at | 9 | CD74 | 127.0 | 2068.6 | 16.3 | 12 |
| 208306_x_at | 11 | HLA-DRB3 | 83.6 | 856.6 | 10.2 | 9, 5*, 11*, 36* |
| 206398_s_at | 12 | CD19 | 18.4 | 412.5 | 22.4 | 8 |
| 214551_s_at | 14 | CD7 | 140.7 | 7.7 | 18.2 | 19, 18* |
| 210116_at | 15 | SH2D1A | 380.5 | 9.0 | 42.1 | 13, 51*, 97* |
| 201137_s_at | 17 | HLA-DPB1 | 55.2 | 894.4 | 16.2 | 1 |
| 211990_at | 18 | HLA-DPA1 | 40.4 | 1009.8 | 25.0 | 15, 16* |
| 209312_x_at | 20 | HLA-DRB3 | 76.6 | 1215.4 | 15.9 | 11, 5*, 9*, 36* |
| **227646_at** | **2** | **EBF** | **10.3** | **253.3** | **24.6** | **23, 24*, 26*** |
| **229487_at** | **4** | **EBF** | **12.9** | **262.3** | **20.4** | **24, 23*, 26*** |
| **50221_at** | **5** | **TFEB** | **18.4** | **40.0** | **2.2** | **-** |
| **221969_at** | **10** | **PAX5** | **7.9** | **216.0** | **27.4** | **22** |
| **232204_at** | **13** | **EBF** | **10.3** | **181.3** | **17.6** | **26, 23*, 24*** |
| **205101_at** | **16** | **MHC2TA** | **13.2** | **41.8** | **3.2** | **-** |
| **215193_x_at** | **19** | **HLA-DRB3** | **65.9** | **1093.7** | **16.6** | **36, 5*, 9*, 11*** |
| **TEL-AML1** |  |  |  |  |  |  |
| 206231_at | 1 | KCNN1 | 166.1 | 51.3 | 3.2 | 15 |
| 241505_at | 2 | --- | 165.3 | 28.7 | 5.8 | 4 |
| 206033_s_at | 3 | DSC3 | 58.9 | 6.7 | 8.8 | 12, 11* |
| 206032_at | 5 | DSC3 | 29.0 | 6.0 | 4.9 | 11, 12* |
| 220451_s_at | 6 | BIRC7 | 59.0 | 14.6 | 4.0 | 16 |
| 204849_at | 7 | TCFL5 | 507.7 | 63.3 | 8.0 | 14, 9* |
| 205109_s_at | 8 | ARHGEF4 | 71.4 | 9.3 | 7.7 | 5, 43* |
| 213558_at | 9 | PCLO | 292.9 | 10.5 | 27.8 | 7, 6* |
| 210650_s_at | 10 | PCLO | 40.9 | 5.8 | 7.6 | 6, 7* |
| 235694_at | 11 | TCFL5 | 58.2 | 12.4 | 4.7 | 9, 14* |
| 228158_at | 14 | --- | 61.2 | 22.4 | 2.7 | 18 |
| 213317_at | 15 | --- | 79.3 | 5.7 | 13.9 | 20 |
| 202808_at | 18 | C10orf26 | 247.5 | 71.5 | 3.5 | 10 |
| **214110_s_at** | **4** | **---** | **21.4** | **9.9** | **2.2** | **-** |
| **219866_at** | **12** | **CLIC5** | **64.8** | **18.0** | **3.6** | **38, 20*, 41*** |
| **213017_at** | **13** | **ABHD3** | **135.1** | **37.8** | **3.6** | **97** |
| **221748_s_at** | **16** | **TNS** | **173.8** | **27.4** | **6.3** | **25, 29*** |
| **204615_x_at** | **17** | **IDI1** | **278.1** | **119.4** | **2.3** | **27, 28*** |
| **221747_at** | **19** | **TNS** | **124.3** | **34.6** | **3.6** | **29, 25*** |
| **203611_at** | **20** | **TERF2** | **1807.2** | **304.7** | **5.9** | **48, 60*, 61*** |

**†**The ranking refers to the top100 subgroup-discriminating probe sets identified by Ross *et al*. using the parallel analysis format.

*Denotes genes that are represented in Ross top 100 (parallel format) by different probe sets (see also Table S2).

Probe sets/ genes not represented in Ross top 20 (parallel format) are printed in bold.
