# Supplementary material for: Translating microarray data for diagnostic testing in childhood leukaemia
Source: BMC Cancer. 2006 Sep 26;6:229. doi: 10.1186/1471-2407-6-229 (PMC1609180; doi:10.1186/1471-2407-6-229)
Supplement: Additional file 2 — Table S2: Common probes sets and genes within the top 20 discriminators per subgroup identified by RMA/RF and Ross et al. (parallel format). [file 1471-2407-6-229-S2.doc]

**Additional file 2**

**Table S2:** Common probes sets and genes within the top 20 discriminators per subgroup identified by RMA/RF and Ross *et al*. (parallel format).

| **Subgroup** | **Common probe sets** | Genes (RMA/RF) | Common genes |
| --- | --- | --- | --- |
| *BCR*-*ABL* | 9 (45%) | 16 | 9/16 (56.3%) |
| *E2A*-*PBX1* | 13 (65%) | 15 | 9/15 (60.0%) |
| Hyperdipl.>50 | 7 (35%) | 20 | 7/20 (35.0%) |
| MLL | 11 (55%) | 17 | 9/17 (52.9%) |
| T-ALL | 13 (65%) | 14 | 10/14 (71.4%) |
| *TEL*-*AML1* | 13 (65%) | 15 | 10/15 (66.7%) |
