# Supplementary material for: Translating microarray data for diagnostic testing in childhood leukaemia
Source: BMC Cancer. 2006 Sep 26;6:229. doi: 10.1186/1471-2407-6-229 (PMC1609180; doi:10.1186/1471-2407-6-229)
Supplement: Additional file 3 — Table S3: Average classification accuracy (100 cross validations) of test set samples (n = 25) using the top 20, 15, 10, 5 and 2 probe sets per subgroup identified by RMA/RF. For each analysis a new training and test set was chosen and discriminating probe sets were identified using the new training set. [file 1471-2407-6-229-S3.doc]

**Additional file 3**

**Table S3:** Average classification accuracy (100 cross validations) of test set samples (n=25) using the top 20, 15, 10, 5 and 2 probe sets per subgroup identified by RMA/RF. For each analysis a new training and test set was chosen and discriminating probe sets were identified using the new training set.

| **Probe sets (total)** | **BCR-ABL (n=4)** | **E2A-PBX1 (n=5)** | **Hyperdip. >50 (n=4)** | **MLL (n=5)** | **T-ALL (n=2)** | **TEL-AML1 (n=5)** | **Overall Accuracy** |
| --- | --- | --- | --- | --- | --- | --- | --- |
| 120 | 89% | 100% | 99.8% | 100% | 100% | 100% | 98.2% |
| 90 | 88.8% | 100% | 99.5% | 100% | 100% | 100% | 98.1% |
| 60 | 89% | 100% | 99.3% | 100% | 100% | 100% | 98.1% |
| 30 | 88.5% | 100% | 99.3% | 100% | 100% | 100% | 98% |
| 12 | 89.3% | 99% | 99% | 99.4% | 98.5% | 98.4% | 97.4% |
