# Supplementary material for: Translating microarray data for diagnostic testing in childhood leukaemia
Source: BMC Cancer. 2006 Sep 26;6:229. doi: 10.1186/1471-2407-6-229 (PMC1609180; doi:10.1186/1471-2407-6-229)
Supplement: Additional file 4 — Table S4: Independent data set of ALL specimens used for verification of subtype classification (n = 68). [file 1471-2407-6-229-S4.doc]

**Additional file 4**

**Table S4:** Independent data set of ALL specimens used for verification of subtype classification (n=68).

| **Sample** | **Source** |
| --- | --- |
| ***BCR-ABL* subgroup (n=3)** | |
| BCR-ABL-1 | BM |
| BCR-ABL-2 | BM |
| BCR-ABL-3 | BM |
| ***E2A-PBX1* subgroup (n=3)** | |
| E2A-PBX1-1 | BM |
| E2A-PBX1-2 | Cell line (PER-278) |
| E2A-PBX1-3 | Cell line (PER-371) |
| **Hyperdip>50 subgroup (n=17)** | |
| Hyperdip>50-1 | BM |
| Hyperdip>50-2 | BM |
| Hyperdip>50-3 | BM |
| Hyperdip>50-4 | BM |
| Hyperdip>50-5 | BM |
| Hyperdip>50-6 | BM |
| Hyperdip>50-7 | BM |
| Hyperdip>50-8 | BM |
| Hyperdip>50-9 | BM |
| Hyperdip>50-10 | BM |
| Hyperdip>50-11 | BM |
| Hyperdip>50-12 | BM |
| Hyperdip>50-13 | BM |
| Hyperdip>50-14 | BM |
| Hyperdip>50-15 | BM |
| Hyperdip>50-16 | BM |
| Hyperdip>50-17 | BM |
| ***MLL* subgroup (n=7)** |  |
| MLL-1 | BM |
| MLL-2 | BM |
| MLL-3 | BM |
| MLL-4 | BM |
| MLL-5 | Cell line (PER-377) |
| MLL-6 | Cell line (PER-485) |
| MLL-7 | Cell line (PER-490) |
|  |  |
|  |  |
|  |  |
|  |  |
|  |  |
| **Sample** | **Source** |
| **T-ALL subgroup (n=37)** | |
| T-ALL-1 | BM |
| T-ALL-2 | BM |
| T-ALL-3 | BM |
| T-ALL-4 | BM |
| T-ALL-5 | BM |
| T-ALL-6 | BM |
| T-ALL-7 | BM |
| T-ALL-8 | BM |
| T-ALL-9 | BM |
| T-ALL-10 | BM |
| T-ALL-11 | BM |
| T-ALL-12 | BM |
| T-ALL-13 | BM |
| T-ALL-14 | BM |
| T-ALL-15 | BM |
| T-ALL-16 | BM |
| T-ALL-17 | BM |
| T-ALL-18 | PB |
| T-ALL-19 | BM |
| T-ALL-20 | PB |
| T-ALL-21 | PB |
| T-ALL-22 | BM |
| T-ALL-23 | Cell line (ALL-SIL) |
| T-ALL-24 | Cell line (CEM-CCRF) |
| T-ALL-25 | Cell line (DU528) |
| T-ALL-26 | Cell line (HSB2) |
| T-ALL-27 | Cell line (Jurkat) |
| T-ALL-28 | Cell line (MOLT4) |
| T-ALL-29 | Cell line (PER-117) |
| T-ALL-30 | Cell line (PER-255) |
| T-ALL-31 | Cell line (PER-427) |
| T-ALL-32 | Cell line (PER-487) |
| T-ALL-33 | Cell line (PER-537) |
| T-ALL-34 | Cell line (PER-550) |
| T-ALL-35 | Cell line (PER-604) |
| T-ALL-36 | Cell line (PER-606) |
| T-ALL-37 | Cell line (PER-608) |
| ***TEL-AML1* subgroup (n=1)** | |
| TEL-AML1-1 | Cell line (PER-145) |
