# Supplementary material for: Translating microarray data for diagnostic testing in childhood leukaemia
Source: BMC Cancer. 2006 Sep 26;6:229. doi: 10.1186/1471-2407-6-229 (PMC1609180; doi:10.1186/1471-2407-6-229)

**Additional file 5**

**Figure S1: Identification of different subgroup-discriminating genes.** Probe sets and genes that were commonly identified as subgroup-discriminators using RMA/RF and as reported by Ross *et al*. (2003). The top 20 discriminators for each of the six ALL subgroup were compared. The percentage of common probe sets is represented by light grey bars, while the percentage of common genes is indicated by dark grey bars.


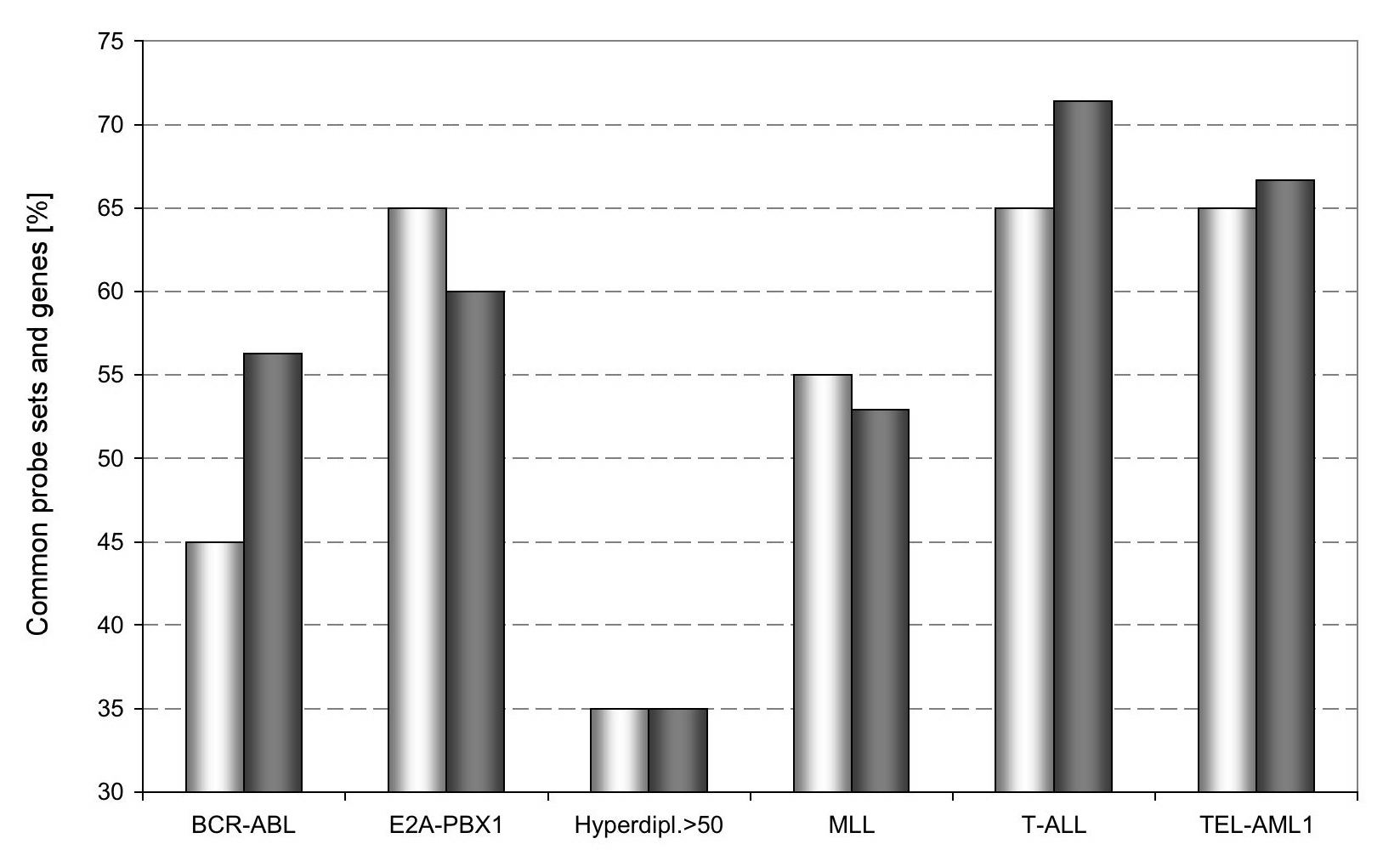

Supplement: Additional file 5 — Figure S1: Identification of different subgroup-discriminating genes. Probe sets and genes that were commonly identified as subgroup-discriminators using RMA/RF and as reported by Ross et al. (2003). The top 20 discriminators for each of the six ALL subgroup were compared. The percentage of common probe sets is represented by light grey bars, while the percentage of common genes is indicated by dark grey bars. [file 1471-2407-6-229-S5.doc]
